# Vibration mitigation efficiency of an inclined, curved, open trench

**DOI:** 10.1371/journal.pone.0229010

**Published:** 2020-02-06

**Authors:** Aneta Herbut

**Affiliations:** Wroclaw University of Science and Technology, Faculty of Civil Engineering, Institute of Geotechnics and Hydrotechnics, Wroclaw, Poland; University of Glasgow, UNITED KINGDOM

## Abstract

The vibration screening efficiency of a curved, inclined, open trench is investigated in this paper. An elastic, transversely isotropic half-space with hysteretic damping, acted upon by a harmonic vertical excitation, is assumed. Equations of motion with absorbing boundary conditions are presented and numerically integrated using FlexPDE software, based on the finite element method. The barrier efficiency is analysed in terms of the reduction of the vertical and horizontal components of the ground surface vibration. The results are presented for trenches with different geometric features based on the non-dimensional amplitude reduction factor. The trench inclination angle, the shape of the trench surface and the distance between the source of vibration and an obstacle are investigated as factors that can improve the trench reduction ability. It is demonstrated that a better attenuation of vibration is achieved with the proposed inclined trench than with its vertical counterpart. Moreover, the vibration mitigation effect is more significant in the case of a more horizontally located trench than for its vertical counterpart (even up to 5 times smaller displacement amplitudes for the vertical displacement component). Additionally, assuming the same trench depth, the vibration reduction effect is better in the case of a smaller number of rings used. The vibration attenuation efficiency is more visible if the trench is located closer to the vibration source.

## 1. Introduction

**Fig 1:**
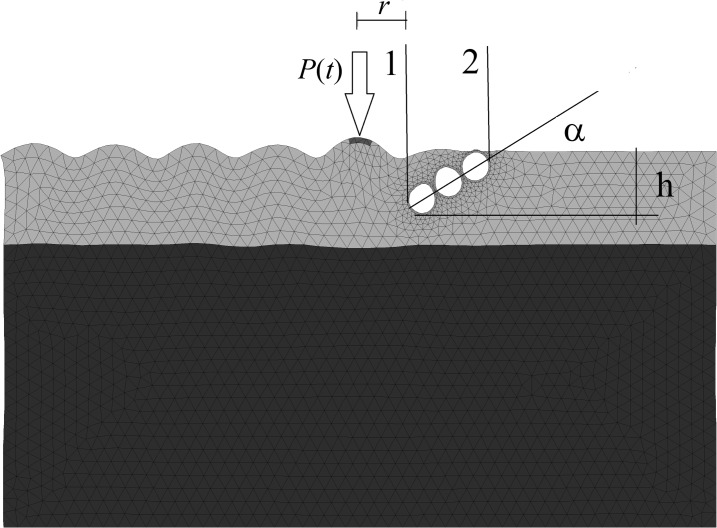
Deformation of the Ground Surface Using an Inclined, Open Trench for *t* = 5T.

Approaches for preventing the failure of structures due to seismic, wind or man-induced vibrations can generally be divided into two groups: the use of excitation-resistant structures and joints based on passive, active or semi-active vibration mitigation techniques [1,2] and the use of barriers in the ground to prevent the transmission of surface wave energy. While the first method has been widely explored in the literature, very few studies have evaluated the second approach, as noted by Kuznetsov [3]. The approach proposed in this paper, based on an inclined, curved, open trench, belongs to the second group. Vibration screening allows for the possibility of preventing energy from reaching sensitive zones. The idea is simple, and if required screening geometry criteria are met, a reliable solution can be obtained. The majority of the energy that affects nearby structures is carried by a Rayleigh wave travelling on a ground surface, away from the source of vibration. According to Towhata [4], this energy comprises approximately 67 per cent of the total energy. A barrier in the form of a ground discontinuity makes wave propagation impossible. A vibration attenuation effect is achieved by the proper interception, scattering and diffraction of surface waves. After incidence on an obstacle, the majority of the energy of the Rayleigh wave is reflected, with partial transmission through the barrier, and new body waves radiate outwards. Two types of waves are generated in this process, reflected waves propagating downwards and on the left side of the barrier and transmitted waves that propagate on the right side of the trench [5]. Different solutions can be implemented to achieve wave screening, including sheet-pile walls, piles [6,7] and trenches filled with water, soft soil, concrete or special materials such as Geofoam [8]. However, the best results are achieved by the use of so-called open trenches [5,9]. Woods [10] first reported results from a wide-field investigation of so-called active and passive isolations by the use of open trenches in 1967. An active isolation is realised by a barrier that surrounds a source of vibration, in which the distance between the exciter and the trench is relatively small. According to Woods, to achieve a satisfactory level of vibration mitigation, an active open trench should be more than 0.6*λ*_R_ in depth, where *λ*_R_ *= V*_R_/*f* is the length of the Rayleigh wave, *V*_R_ is the Rayleigh wave velocity and *f* is the excitation frequency. Woods considered barriers located 0.222*λ*_R_-0.910*λ*_R_ from the source of vibration. In contrast, passive isolation involves a trench that is distant from the source of disturbance, located near a point at which the vibrations must be reduced. The distance between the source of vibration and the centre of the trench must equal 2*λ*_R_-7*λ*_R_. In this case, to achieve satisfactory vibration attenuation, the trench depth must be approximately 1.2*λ*_R_-1.5*λ*_R_. In both cases, the trench width has practically no influence on the isolation efficiency [10]. Shrivastava and Rao [5] analysed the effectiveness of open and filled trenches for screening Rayleigh waves. The authors considered an impulse-excited vibration. The authors reached the same conclusion as Woods [10]–an open trench is always a better solution than an in-filled trench. The depth of the trench appears to be the most important parameter, and the width has little effect on the isolation efficiency, except for very narrow trenches. Similar conclusions were presented by Çelebi et al. [9], who completed broad experimental investigations on the influence of trench filling on screening efficiency in the case of a harmonic excitation. The use of an open trench is more effective than using an in-filled trench, but its practical application is limited to relatively shallow depths. However, using a softer backfill increases the effectiveness of the in-filled trench and allows for a larger trench depth with no need for support measures for the vertical wall. Full-scale field experimental studies were also presented by Alzawi et al. [8] on the protective performance of both open trenches and those in-filled with Geofoam material. A Geofoam barrier can be considered a practical alternative for wave scattering. Studies on the efficiency of a pair of in-filled trenches against harmonic excitation have been reported as well [11]. Kattis et al. [6] compared the screening efficiency for a concrete trench and a row of piles against harmonic excitation. The main conclusion of these investigations is that a trench, especially one that is open, is more efficient than a row of piles. The shape of the piles (circular or square cross-section) does not significantly influence the solution. The screening efficiency of rows of piles and its dependence on the pile geometry and location were also analysed by Gao et al. [7]. Turan et al. [12] analysed the vibration reduction efficiency of an inclined secant micro-pile wall positioned as an active vibration barrier. Among other conclusions, the authors indicate that inclined secant micro-pile walls used as active vibration barriers perform better than their vertical counterparts. A wave scattering effect, similar to that of wave barriers, can be obtained by landscapes shaped in the proper way. A special arrangement of hills and valleys results in Rayleigh wave mitigation of up to 35% of the initial values of ground surface displacements [13]. However, this solution is rather limited to the case of small values of the Rayleigh wavelength (high excitation frequency and/or soft soils). This limit can be overcome by an inclined, open trench, which is presented in this paper (Fig 1). It avoids slope instability problems, and in that way, it can be used for a wider range of ground or load conditions.

The new up-to-date approach is to use metamaterials and metabarriers for the attenuation of ground vibrations. Metamaterials are innovative composite materials with a periodic structure consisting of inclusions placed in the matrix. The idea comes from phononic/photonic crystals. These new materials exhibit untypical features through contact with electromagnetic or elastic waves. We are able to control the elastic wave propagation by the proper selection of material parameters (elastic moduli). Three different strategies of wave manipulation appear in the case of metamaterials, local resonances, Bragg scattering and an invisibility cloak [14]. A similar solution to classical soil trenches in soil is to use metabarriers below the ground surface for seismic protection. Metabarriers are special structures surrounding vibration sensitive areas. Due to the periodic arrangement of inclusions in the matrix, it is possible to attenuate the elastic wave energy by an energy scattering effect [15–17] or by energy dissipation during resonant vibrations of inclusions [18]. Inclusions in metabarriers are usually placed in the soil matrix as columns (are vertically orientated). They can be composed of steel or/and concrete [17]. Empty holes can also be used [15]. The curved inclined, open trench proposed in the paper is composed of inclusions in the soil matrix too. However, the orientation of inclusions is opposite that of metabarriers; here, they are horizontally located in the soil (parallel to the ground surface) (Fig 1). However, in both cases, the effect of wave energy scattering is achieved.

In summary, the aim of this paper is to present a more efficient version of an open trench. As mentioned above, an open trench is a better solution for vibration screening than its filled counterparts or a row of piles [5,10], which is why this type of barrier is studied in the paper. The new idea is based on an inclined, open trench, as inclination of the trench can make the vibration mitigation effect more significant [12]. Moreover, it is shown in the paper that a curved trench surface can also improve the effect of wave energy scattering. The obstacle in the form of an inclined trench is composed of concrete and empty rings (Fig 1). In the paper, the vibration mitigation efficiency in relation to the trench inclination, the shape of its surface and the distance between the exciter and the barrier is investigated. It is proven that the proposed version of an inclined, curved open trench can be more efficient than the classical vertical open trench. Moreover, through the use of the proposed solution, slope instability problems of a classical open trench can be overcome.

## 2. Mathematical model

### 2.1. Equations of motion for a transversely isotropic medium with hysteretic damping

To obtain a correct model of the soil medium, an estimation of strains is necessary. For a relatively large strain (a shear strain larger than 10^−4^), a two-phase elastic-plastic soil medium model based on the Biot theorem is necessary [19]. For the analyses of the surface wave propagation in a soil medium, a one-phase elastic soil model is usually applied [5,11,12].

Let us consider the stresses acting on a soil element with side measurements d*x*, d*y*. The sum of forces acting in the *x* and *y* directions, taking the damping forces into account, gives the differential equations of motion for the soil medium [19–21].

$$\begin{array}{c} \sum P_{x}=0:\;\frac{\partial \sigma_{x}}{\partial x}+\frac{\partial \tau_{yx}}{\partial y}+tr∙\frac{\partial \sigma_{x}}{\partial t\partial x}+tr∙\frac{\partial \tau_{yx}}{\partial t\partial y}=\rho \frac{\partial^{2}u}{\partial t^{2}}\\ \sum P_{y}=0:\;\frac{\partial \sigma_{y}}{\partial y}+\frac{\partial \tau_{xy}}{\partial x}+tr∙\frac{\partial \sigma_{y}}{\partial t\partial y}+tr∙\frac{\partial \tau_{xy}}{\partial t\partial x}=\rho \frac{\partial^{2}v}{\partial t^{2}} \end{array}$$ (1)

where *u*, *v* are the components of displacement in the *x* and *y* directions respectively; *ρ* denotes the soil density; *σ*_*x*_, *σ*_*y*_ *τ*_*xy*_ *= τ*_*yx*_ are the normal and shear stresses, respectively; and *tr* is the relaxation time, which is inversely proportional to the excitation frequency *tr* = 2*ξ*/*ω*. The relations for the strains in terms of the displacements are assumed to be *ε*_*x*_ = ∂*u*/∂*x*, *ε*_*y*_ = ∂*v*/∂*y*, *γ*_*xy*_ = ∂*v*/∂*x+*∂*u*/∂*y*. For an elastic transversely isotropic material, the strain-stress relationship can be presented as [22]

$$\left\{\begin{array}{c} \varepsilon_{x}\\ \varepsilon_{y}\\ \gamma_{xy} \end{array}\right\}=\left[\begin{array}{ccc} {1/E}_{x} & -\nu /E_{y} & 0\\ -\nu /E_{y} & {1/E}_{y} & 0\\ 0 & 0 & 1/G_{xy} \end{array}\right]\left\{\begin{array}{c} \sigma_{x}\\ \sigma_{y}\\ \tau_{xy} \end{array}\right\}$$ (2)

where *E*_*x*_, *E*_*y*_ are the Young’s modulus in the *x* and *y* directions, respectively; *ν* is the Poisson ratio; and *G*_*xy*_ is the shear modulus.

### 2.2. Boundary conditions and finite element model

To avoid wave reflection at the boundary, viscous boundary conditions are assumed, following Lysmer and Kuhlemeyer [23]. The normal *σ* and shear *τ* stress components in virtual dampers, “fixed” to the right and left boundaries (Fig 1), are given by the formulas

$$\sigma_{x}=a\rho V_{x}\dot{u},\;\tau_{xy}=b\rho V_{xy}\dot{v},$$ (3)

where is the mass density of the soil; $\dot{u}$ and $\dot{v}$ are the velocities in the *x* and *y* directions, respectively and *a*, *b* are the wave relaxation coefficients introduced to improve the wave absorption at the boundaries in the normal and tangential directions, respectively. Research findings indicate that *a* = 1 and *b* = 0.25 give reasonable absorption at the boundary [12,24]. These values are also assumed in the present study. The notation *V*_*ij*_ indicates that wave propagation velocity is in the *i*-direction, while the polarisation is in the *j*-direction. An explanation of the phenomenon of wave propagation for different types of anisotropy, including the determination of wave velocities, is omitted here but has been widely discussed in the literature [22,25]. The velocities in the plane of isotropy of the transversely isotropic medium are defined as $V_{xy}=\sqrt{G_{xy}/\rho },\;V_{x}=V_{xx}=\sqrt{E_{x}/\rho }$. In the plane perpendicular to the plane of isotropy, the velocities can be calculated from the following formulas: $V_{yx}=\sqrt{G_{xy}/\rho },\;V_{y}=V_{yy}=\sqrt{E_{y}/\rho }$.

Similarly, the normal *σ* and shear *τ* stress components in virtual dampers “fixed” to the bottom boundary are given by the formulas

$$\sigma_{y}=a\rho V_{y}\dot{v},\;\tau_{yx}=b\rho V_{yx}\dot{u}.$$ (4)

Both displacement components are assumed to be zero at the bottom edge of the investigated region.

An oscillatory load is applied to a square 2 m/0.5 m concrete plate

$$P(t)=Asin(2\;\pi \;f∙t)∙(H(t-t_{b})-H(t-t_{e})),$$ (5)

where *A* is the excitation amplitude, *f* is the excitation frequency, H(*t*) is the Heaviside function, *t*_b_ is the time at which the generator starts to work, and *t*_e_ is the time at which the generator finishes working. For the analysed example, *t*_b_ = 0, *t*_e_ = 5T, where *T* is the period of vibration.

The presented partial differential equations with the corresponding boundary conditions are solved using FlexPDE Professional Version 5.0.7. software based on the finite element method. Triangular finite elements are assumed. The maximum size of the element is limited, according to Kramer, to 1/8 of the minimum wavelength considered in the analyses [23,26]. A fully bonded soil-foundation interface is assumed. The model consists of 7161 nodes and 28644 unknowns. To validate the finite element model, convergence studies have been performed with respect to number of elements assumed. The maximum observed displacement amplitude on the right side of the analysed region for the assumed number of elements was compared to the case of very dense FEM mesh (93394 nodes, 373576 unknowns assumed). For the assumed number of elements, the relative error was approximately 0.1% and less than 0.2% for displacement components in the *x*- and *y*-direction, respectively.

A transversely isotropic, layered half-space is assumed (Fig 1). For a shallow deposit, the dynamic soil parameters are as follows: the shear modulus *G*_*xy*_ = 125 MPa, the Young’s modulus in the plane of isotropy *E*_*x*_ = 3.865*G*_*xy*_, the Young’s modulus in the plane perpendicular to the plane of isotropy *E*_*y*_ = 2.863*G*_*xy*_, the Poisson ratio *ν* = 0.301, the mass density of the soil *ρ* = 2000 kg/m^3^, and the damping coefficient *ξ* = 1% [27]. For a deeper deposit, *G*_*xy*_ = 200 MPa, *E*_*x*_ = 3.865*G*_*xy*_, *E*_*y*_ = 2.863*G*_*xy*_, *ν* = 0.301, *ρ* = 2000 kg/m^3^, and *ξ* = 1%. For the concrete plate and rings, the elastic isotropic material model with the following dynamic parameters is assumed: *G*_*xy*_ = 12.2 GPa, *E*_*x =*_ *E*_*y*_ *=* 27 GPa, *ν* = 0.167, and *ρ* = 2500 kg/m^3^ [19]. The excitation amplitude *A* = 1 kN/m^2^, and the excitation frequency *f* = 30 Hz. The inclined trench is composed of concrete rings, 15 cm thick, contacting each other (Fig 1). The depth of the trench *h* = 0.7*λ*_R_, and the distance between the centre of the applied load and the trench in the *x*-direction *r* = 0.*5λ*_R_ in each analysed case, except in the examples for which this value is considered to vary.

## 3. Results of numerical analyses

**Fig 2:**
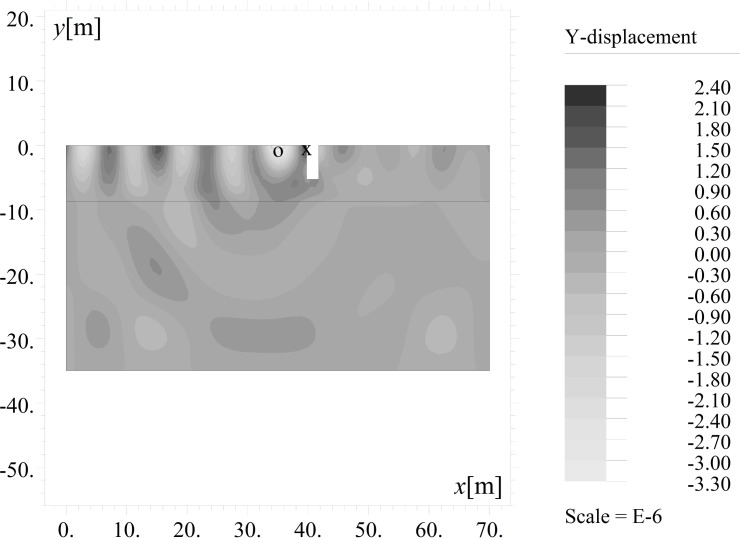
Vertical Displacement Component *v*[m] with the Use of the Open Trench (Point 1) for *t* = 5T.

**Fig 3:**
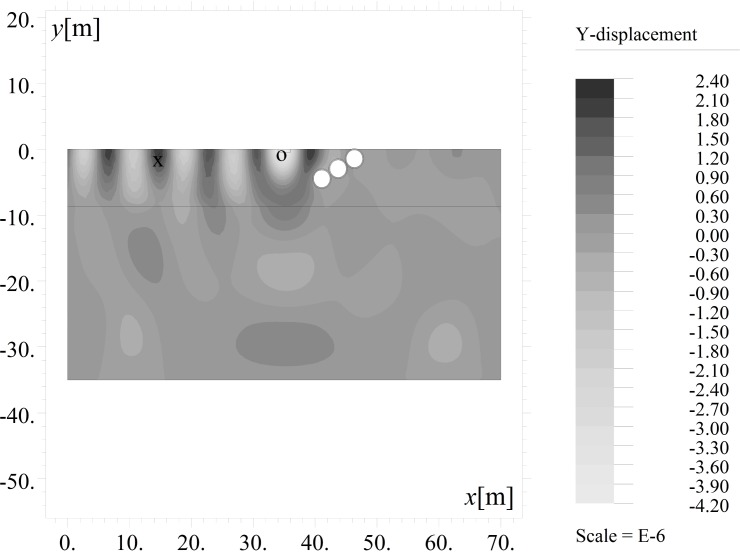
Vertical Displacement Component *v*[m] with the Use of the Inclined, Open Trench for *t* = 5T.

**Fig 4:**
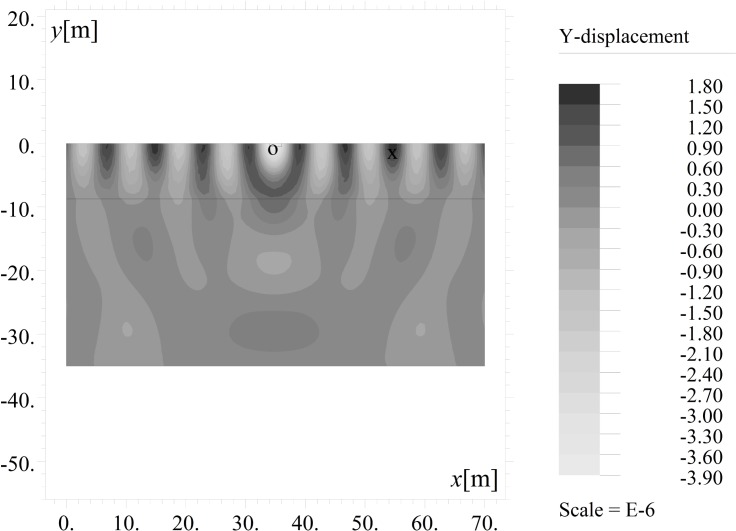
Vertical Displacement Component *v*[m] without the Trench for *t* = 5T.

The results are presented in the form of the amplitude mitigation factor (AMF), defined according to Woods [10] as the ratio between the maximum absolute value of the vertical (AMF_*y*_) or horizontal (AMF_*x*_) displacement observed after using the trench (*v*, *u*) (Figs 2 and 3) and the maximum displacement for the same points without the trench (*v*_o_, *u*_o_) (Fig 4). The amplitude mitigation factor for the vertical displacement component (AMF_*y*_) can be presented by the following formula:

$${AMF}_{y}=Max(Abs(v))/Max(Abs(v_{o})).$$ (6)

A similar definition can be introduced for the horizontal displacement component (AMF_*x*_). In each presented case, analyses are conducted for the area on the right side of the trench (Fig 4), for twenty uniformly spaced points on the ground surface. The displacements are evaluated over 5 time periods for 159 points in time. The maximum absolute value at each point for both vibration components is established.

### 3.1. Vibration attenuation efficiency of an inclined, open trench compared to that of its vertical counterpart

**Fig 5:**
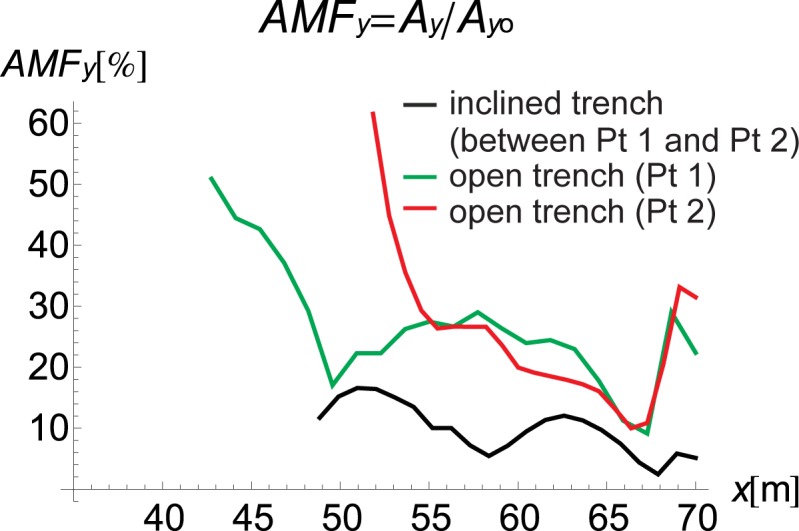
AMF for the Vertical Displacement: Red–Open Trench (Point 2), Green–Open Trench (Point 1), and Black–Inclined Trench *α* = 30° (between Points 1 and 2).

**Fig 6:**
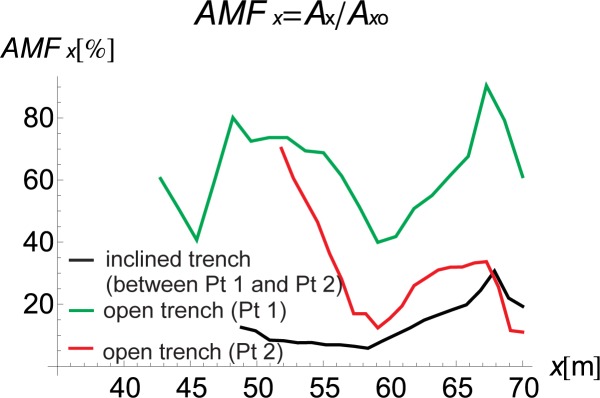
AMF for the Horizontal Displacement; Red–Open Trench (Point 2), Green–Open Trench (Point 1), and Black–Inclined Trench *α* = 30° (between Points 1 and 2).

The inclined, open trench reduces the amplitudes of both vertical and horizontal displacements (Figs 2 and 4–6). In Figs 5 and 6, the results for the right side of the trench are presented for three cases in the form of the dimensionless amplitude mitigation ratio AMF (6)–two vertical open trenches (as in Fig 3) and an inclined, open trench composed of three concrete rings (as in Fig 4). In each analysed case, the depth of the barrier is the same, but the vertical open trenches have different positions; one is located at point 1 (see Fig 1) at the beginning of the inclined trench close to the vibration generator, which is denoted by the green line in Figs 5 and 6. The second is located at point 2 near the point at which the inclined trench reaches the ground surface (Fig 1), as denoted by the red line in Figs 5 and 6. The inclined trench is located between points 1 and 2 (Fig 1)–shown by the black line in Figs 5 and 6. The aim of the analyses is to compare the amplitude reduction efficiency for the two configurations: the inclined barrier and the rectangular vertical trench. It is seen that for both locations of the classical vertical open trench, the AMF ratios are greater than those of the proposed inclined, open trench. This finding holds for both displacement components, demonstrating that the reduction ability is better for the inclined trench than for the vertical trench. The reduction effect is more visible in the area close to the barrier and closer to the excitation force (Figs 5 and 6). The closer the distance to the vibration source, the greater the values of the displacement amplitudes. An important conclusion is that a significant improvement in the reduction effect can be observed in the area where the displacements have the greatest values.

### 3.2. The shape of the trench and the screening efficiency

**Fig 7:**
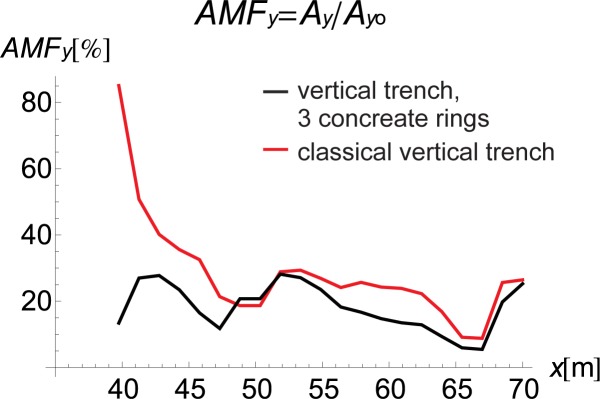
AMF for the Vertical Displacement: Black–Vertical Trench, 3 Concrete Rings, *α* = 90° and Red–Classical Vertical Trench.

**Fig 8:**
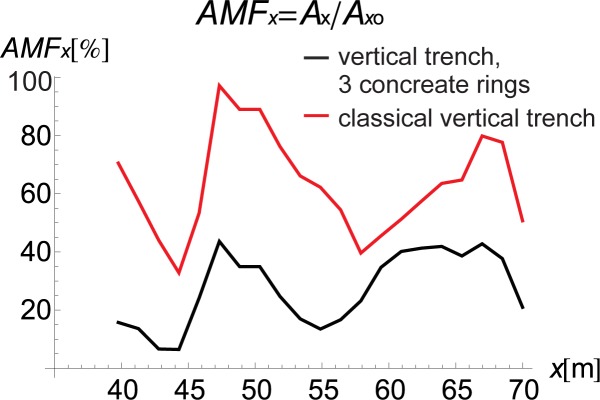
AMF for the Horizontal Displacement: Black–Vertical Trench, 3 Concreate Rings, *α* = 90° and Red–Classical Vertical Trench.

The curved shape of the proposed inclined trench (Fig 4) is used instead of the straight surface of the classical open trench (Fig 3) to provide for implementation of the solution. Due to slope instability problems, it is obviously not possible to build an inclined trench in the soil. Additionally, the curved shape improves the effect of the vibration attenuation of the barrier, as observed in Figs 7 and 8. In these figures, the AMF ratios on the right side of the trench are presented for two vertical trenches. The first trench is the classical open trench, with a rectangular shape (red lines in Figs 7 and 8). The second barrier is the vertical trench, which is composed of 3 rings (*α* = 90° in Fig 1) (black lines in Figs 7 and 8). The trench depth and the distance between the source of vibration and the obstacle are the same for both of the considered cases (*h* = 0.7*λ*_R_ and *r* = 0.5*λ*_R_). It can be observed that the vibration attenuation effect is stronger for the trench consisting of rings. This result is more visible for the horizontal component of the surface wave than for the vertical component (Figs 7 and 8). For the vertical vibration component, the reduction in displacement is more significant when it is closer to the vibration source.

**Fig 9:**
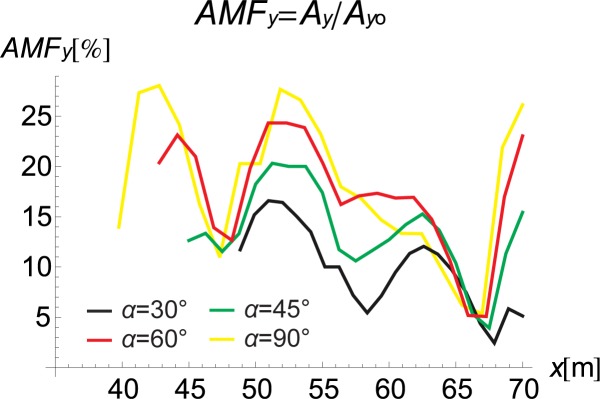
AMF for the Vertical Displacement: Yellow–*α* = 90°, Red–*α* = 60°, Green–*α* = 45°, and Black–*α* = 30°.

**Fig 10:**
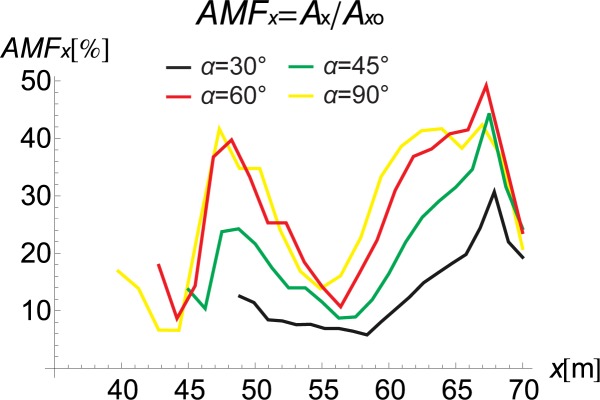
AMF for the Horizontal Displacement: Yellow–*α* = 90°, Red–*α* = 60°, Green–*α* = 45°, and Black– *α* = 30°.

**Fig 11:**
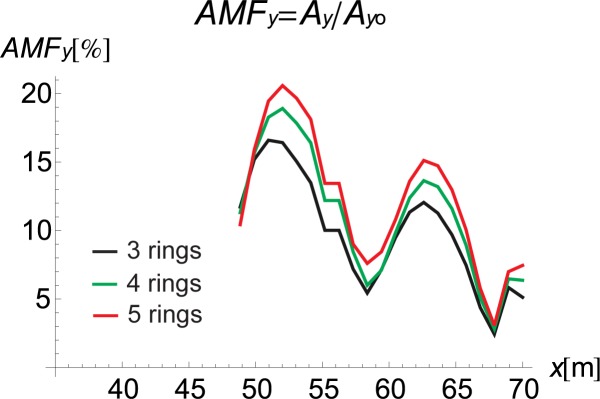
AMF for the Vertical Displacement *α* = 30°: Red–5 Rings, Green–4 Rings, and Black–3 Rings.

**Fig 12:**
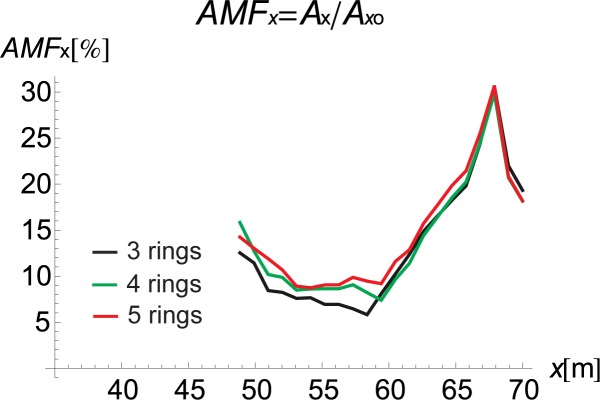
AMF for the Vertical Displacement *α* = 30°: Red–5 Rings, Green–4 Rings, and Black–3 Rings.

If the inclination of the trench and its curved shape improve the vibration reduction, the question remains as to how one should arrange the rings to obtain the optimal reduction effect. For this purpose, the influence of the inclination angle (*α* in Fig 1) and the number of rings on the displacement amplitudes is investigated. It can be observed that the best vibration attenuation effects are obtained for more horizontally orientated barriers (Figs 9 and 10, S1 Dataset) composed of a smaller number of rings (Figs 11 and 12). The inclination of the curved trench gives a better wave reflection than the rectangular open trench as seen from the vibration amplitudes on the left side of the trench (Figs 3 and 4). In the case of the inclined trench, the values of the displacements are greater on the left side of the trench than those in the case of the vertical trench. In addition, less energy is transmitted through the inclined barrier. The vibration amplitude on the right side of the trench decreases more significantly in the case of an inclined trench (Figs 3 and 4). Turan observed similar effect of the inclination for pile walls [12]. Modifying the obstacle shape by using fewer rings, thus obtaining a more curved trench shape also improves the diffraction and scattering effects.

### 3.3. The trench location and the vibration attenuation effect

**Fig 13:**
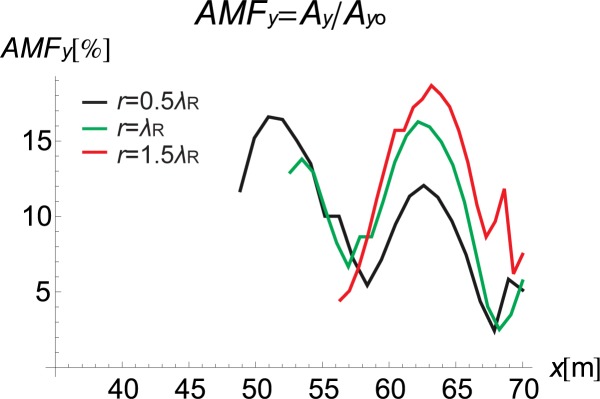
AMF for the Vertical Displacement *α* = 30°: Red–*r* = 1.5*λ*_R_, Green–r = *λ*_R_, and Black–*r* = 0.5*λ*_R_.

**Fig 14:**
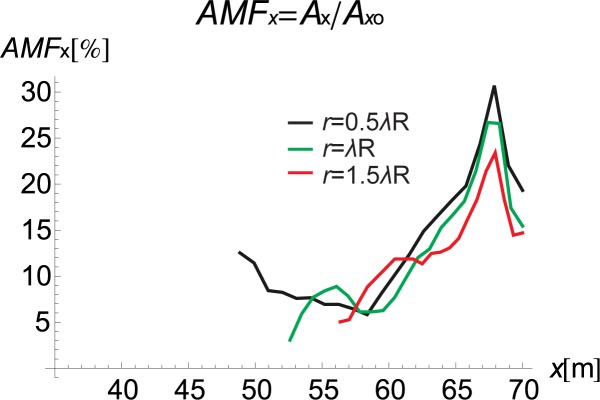
AMF for the Horizontal Displacement *α* = 30°: Red–*r* = 1.5*λ*_R_, Green–*r* = *λ*_R_, and Black–*r* = 0.5*λ*_R_.

The trench location and its influence on the vibration reduction are also analysed. Figs 13 and 14 present the AMF for the right side of the trench for the following three different barrier locations: *r* for half the Rayleigh wavelength, for the Rayleigh wavelength and for one and a half times the Rayleigh wavelength. For the vertical component, a better vibration reduction effect can be observed when the barrier is located closer to the source of vibration, which indicates active isolation rather than passive isolation. This finding is in contrast to the solutions obtained for the horizontal component, where slightly better attenuation of displacements is obtained in the case of passive isolation.

## 4. Discussion

As in the case with earthquake-induced vibrations, wave mitigation techniques are of great importance when manmade vibrations are concerned. Vibrations generated by machines, traffic or geotechnical works can be destructive to both structures and people [28–30]. Hence, the development of vibration reduction methods is of considerable significance. The approach that focuses on improving the dynamic resistance of structures (by applying active, semi-active, or passive dampers to those structures) has been dominant in the protection of structures from dynamic impacts [31–33]. However, at present, vibration mitigation techniques based on the transmission paths of waves are being intensively developed [13,15–18]. The principle of the latter technique is to attenuate the amplitudes of waves during their propagation in soil media; in this way, both individual buildings or structural elements and whole regions comprising many structures can be protected against dynamic impacts. Moreover, with this technique, structural interfere (which is often expensive and not always possible) is not necessary. In the case of existing structures, when additional loads resulting from the application of vibration mitigation devices and from the modification of structural operating conditions are not considered during the design process, reducing the amplitudes of waves in soil may represent the only solution for protecting structures against dynamic impacts. This restriction constitutes the main advantage of the abovementioned methods based on wave attenuation technologies relative to dampers.

In addition, the use of vibration screening, especially in the form of open trenches, is a simple, reliable and relatively cheap method for protecting structures and sensitive equipment from excessive vibrations. However, this concept has also limitations, including the fact that achieving acceptable results requires that the trench depth be relatively large, that is, more than 60% of the Rayleigh wavelength (according to Woods [10]). Unfortunately, the Rayleigh wavelength in soil can exceed 100 m; hence, a classic open trench is usually employed in cases with small values of the Rayleigh wavelength (soft soils with a relatively high excitation frequency). Nevertheless, an open trench is always a better solution for vibration screening than its filled counterpart [5,9,10], which is why this type of barrier is studied herein. In the paper, an improved version of the classic open trench that is composed of many empty rings is proposed. The special composition of this barrier avoids slope instability problems that often appear in the case of a classic open trench. Consequently, the proposed version of an open trench can be extended deeper below the ground surface than a classic open trench and thus can be much more effective [10]. Moreover, the surface of the barrier considered in the paper is curved and inclined, which also improves the wave energy scattering effect in comparison with the classic rectangular open trench.

## 5. Conclusions

In the paper, an improved version of the classical open trench is proposed. The proposed idea of an inclined curved, open trench can be applied to larger trench depths without requiring support, and soil instability problems of the trench walls do not appear, which is the main advantage of the proposed solution. As proven in the paper, this approach is also more efficient than the classical rectangular open trenches. Near the barrier, the vibration reduction efficiency of an inclined, curved, open trench is more than 5 times better than that of a classic rectangular open trench. This beneficial effect becomes more visible closer to the barrier and the vibration source, that is, in the areas where the vibration amplitudes are the largest. This effect can be observed on both the vertical and the horizontal displacement components. Moreover, the proposed method displays better vibration reduction results for both the horizontal and vertical components of the vibration if the trench is relatively horizontal (having a smaller value of the inclination angle, *α*). In addition, assuming the same trench depth, the vibration mitigation effect is better for a barrier composed of a smaller number of rings. The vibration attenuation effectiveness is especially visible for the vertical component of the vibration. The presented solution gives better results for active (a trench located closer to the source of vibration) than for passive isolation.

## Supporting information

S1 Dataset (NB)
